# Deafblindness in Aotearoa, New Zealand: Prevalence and Equity Challenges From a Cross-Sectional Census Analysis

**DOI:** 10.1177/10105395261456806

**Published:** 2026-06-15

**Authors:** Sally Britnell, Nick Garrett, Claire Bretherton

**Affiliations:** 1 School of Nursing, Faculty of Health and Environmental Sciences, Auckland University of Technology, New Zealand; 2 School of Interprofessional Health Studies, Faculty of Health and Environmental Sciences, Auckland University of Technology, New Zealand; 3 Data and Insights, Whaikaha – Ministry of Disabled People, Wellington, New Zealand

**Keywords:** public health, deafblindness, dual sensory impairment, New Zealand, prevalence

## Abstract

Deafblindness or difficulty seeing and hearing even with correction or hearing aids affects an estimated 7.4% (about 295 000) of New Zealanders, with prevalence rising sharply among older adults. Analysis of 2023 Census data using the Washington Group Short Set (WGSS) highlights not only the scale of this issue but also its inequitable distribution: gender-diverse people, Māori, Pacific peoples, and rural communities experience higher rates and earlier onset of sensory difficulties. However, the WGSS’s Western, individualistic framing often undercounts deafblindness in Indigenous and collectivist cultures, risking invisibility for those most affected. Geographic disparities further compound inequity, with rural regions facing greater barriers to care. These findings reveal that current measurement tools and policies may not fully capture the lived realities of deafblind people, limiting access to services and perpetuating disadvantage. Addressing these gaps requires culturally responsive data collection, co-design with affected communities, and integrated, holistic support systems. Recognising deafblindness as a distinct disability and aligning policy with Te Tiriti o Waitangi and the United Nations Convention on the Rights of Persons with Disabilities are essential steps towards equity and inclusion.

## What We Already Know

- Deafblindness (dual sensory impairment) is associated with increased social exclusion, mental health issues, and health care barriers.
- International prevalence varies because of inconsistent definitions and measurement tools.
- The Washington Group Short Set (WGSS) is a common tool for disability data but may not effectively identify deafblindness, particularly in diverse or Indigenous populations.

## What This Article Adds

- Provides the first national estimate of deafblindness in Aotearoa New Zealand using 2023 Census data and WGSS thresholds, identifying 7.4% of the population (≈295,000 people) and highlighting demographic disparities.
- Demonstrates that current self-report measurement approaches can undercount deafblindness, limiting accurate understanding, service eligibility, and effective planning.
- Argues for recognising deafblindness as a distinct disability in policy and calls for culturally responsive, equity-focused approaches aligned with Te Tiriti o Waitangi and the UNCRPD.

## Introduction

Deafblindness (dual sensory impairment; DSI) is more than a clinical label; it is a complex, intersectional, and under-recognised health issue that shapes how people communicate, move through their communities, and access health care. Yet, in Aotearoa, New Zealand, and globally, it is often invisible in population data and policy, leaving many with unmet needs.^1-5^

International prevalence estimates for deafblindness vary widely, reflecting differences in definitions, thresholds, and data sources.^5,6^ Recent global mapping work highlights substantial regional variation in age-related dual sensory impairment, underscoring the need for robust, comparable data.^6^

In Aotearoa, New Zealand, a lack of reliable, equity-focused prevalence data limits planning for accessible services for deafblind people and contributes to unmet need, particularly for communities already experiencing health inequities.^7,8^

Deafblindness is associated with a higher risk of social exclusion, reduced autonomy, poorer mental health, and functional decline.^3-5^ However, as long as people with deafblindness remain unidentified, their complex needs will remain inadequately addressed. Improved data collection is needed internationally to inform equitable service planning and policy response. The Washington Group Short Set (WGSS), while widely used for international disability monitoring, particularly across culturally diverse populations and in contexts where collective support can mask functional limitations, has been the subject of debate leading to questioning of the WGSS’s ability to detect functional sensory difficulties, especially in population-based surveys.^8,9^

Deafblindness, despite its significant impact, is still not sufficiently recognised within public health systems. Until individuals with deafblindness are properly identified, their complex needs will continue to be inadequately met. There is a need for better data collection worldwide to support fair service planning and policy-making. This research examined the prevalence of deafblindness and its distribution in Aotearoa, New Zealand, using 2023 Census data^7^ that included WGSS questions.^8^ In addition, it critically assessed the WGSS’s effectiveness and limitations of WGSS-based identification for equity-focused disability monitoring and public health service planning. The WGSS was chosen because the Census is the only near-universal dataset that includes WGSS items, enabling national estimates and subgroup comparisons not feasible via clinical datasets.

## Methods

This study examined 2023 Aotearoa, New Zealand Census data, which included approximately 4.7 million respondents. Inclusion criteria required completion of both the seeing and hearing questions from the WGSS. The final sample comprised 4 705 542 respondents.

In international studies, deafblindness is typically identified using self-report functional measures, clinical assessments, or administrative records. The WGSS is widely used for disability monitoring, but its suitability for identifying deafblind, particularly across culturally diverse populations, has been debated.^9,10^

The 2023 New Zealand Census included the WGSS questions^8^ on seeing and hearing. The seeing question was ‘Do you have difficulty seeing?’ and the hearing question was ‘Do you have difficulty hearing?’ It was clearly stated that this applied while wearing corrective lenses or hearing instruments. For both questions, response options were: No difficulty (normal vision), Some difficulty, A lot of difficulty, Cannot do at all, and Not Elsewhere Included (NEI) or did not answer. For this analysis, individuals reporting ‘some difficulty’ or more in both seeing and hearing were classified as experiencing deafblindness. Not Elsewhere Included responses were excluded from prevalence estimates.

### Statistical Analysis

Descriptive prevalence estimates were calculated for deafblindness and for individual seeing and hearing difficulties, stratified by age group, gender identity, ethnicity, and District Health Board (DHB) region. Groups with 1 to 6 responses were suppressed (set to 3) to protect anonymity, and NEI responses were excluded from all analyses. Results are presented as proportions and counts, with cross-tabulations by demographic and geographic variables.

#### Accessibility Limitation

Although the census was available in multiple formats, including large print and online, it remains unclear whether all accessibility needs of people with sensory difficulties were fully met. This may have contributed to the underrepresentation of people with deafblindness in the final sample and should be considered a study limitation.

## Results

This study found that 7.4% of people in New Zealand (about 295 000) have difficulty with both seeing and hearing. In this analysis, ‘blind’ refers to respondents who selected ‘cannot do at all’ for seeing, and ‘severe impairment’ refers to those reporting ‘a lot of difficulty’.

Respondents who answered both WGSS seeing and hearing questions were included in the analysis. To protect privacy, groups with 1 to 6 responses were suppressed (set to 3), and NEI responses were excluded, which likely deafblindness, especially given the communication barriers individuals face. The final sample comprised 4 705 542 people.

### Difficulty Seeing and Hearing

Difficulty seeing and hearing are closely linked, with deafblindness increasing as impairments worsen. Among those with some difficulty seeing, 37.4% are deafblind; this rises to 51.5% with a lot of difficulty and 64.9% among those who cannot see. Hearing follows a similar pattern, with deafblind prevalence increasing from 52.1% (some difficulty) to 70.4% (cannot hear), indicating a cumulative effect (Table 1).

**Table 1.** A Breakdown of People’s Reported Hearing and Vision Difficulty Levels.

Difficulty seeing	Difficulty hearing											Deafblind	
	None		Some		A lot		Cannot do		NEI	Total			
	n	(%)	n	(%)	n	(%)	n	(%)	n	n	%	n	(%)
None	2 936 508	(86.3)	242 913	(49.5)	18 507	(30.0)	1269	(23.7)	10 665	3 209 862	(80.5)		
Some	431 934	(12.7)	226 971	(46.3)	29 517	(47.9)	1236	(23.1)	5838	695 496	(17.4)	257 724	(37.4)
A lot	30 855	(0.9)	19 431	(4.0)	12 981	(21.1)	390	(7.3)	1143	64 800	(1.6)	32 802	(51.5)
Cannot do at all	2187	(0.1)	978	(0.2)	597	(1.0)	2463	(46.0)	897	7122	(0.2)	4038	(64.9)
NEI	6630		3942		1041		375		716 274	728 262			
Total	3 408 114	(85.8)	494 235	(12.4)	62 643	(1.6)	5733	(0.1)	734 817	4 705 542		294 564	(7.4)

Abbreviation: NEI, not elsewhere included.

Percentages exclude NEI in calculations.

### Age Patterns

While deafblindness affects 7.4% of New Zealanders, it rises to 21.6% in those over 75. Both hearing and vision difficulties increase with age: hearing problems rise from 3.3% in children to 45% in those over 75, while vision issues increase from 5.7% to 35.3%. Severe impairments also grow significantly with age, highlighting the cumulative effect of ageing on sensory health (Table 2).

**Table 2.** Demographic Data Relating to Difficulty Hearing and Seeing.

					Difficulty hearing				Difficulty seeing			
			Deafblind		None	Some	A lot	Cannot do	None	Some	A lot	Cannot do
Age group (years)	No.	%	n	%	%	%	%	%	%	%	%	%
5-14	647 910	(13.8)	5292	(0.8)	96.7	2.9	0.3	0.1	94.2	5.1	0.5	0.1
15-29	968 298	(20.6)	19 926	(2.1)	94.3	5.2	0.4	0.1	85.8	12.8	1.2	0.1
30-44	1 035 375	(22.0)	23 166	(2.2)	93.7	5.7	0.5	0.1	87.5	11.4	1.0	0.2
45-59	928 938	(19.7)	71 448	(7.7)	85.3	13.3	1.2	0.2	73.1	25.0	1.7	0.2
60-74	762 360	(16.2)	96 240	(12.6)	73.2	23.9	2.8	0.2	72.3	25.4	2.1	0.2
75+	362 670	(7.7)	78 492	(21.6)	55.0	37.1	7.6	0.3	64.7	29.7	5.1	0.5
Gender	No.	%	n	%	%	%	%	%	No.	%	n	%
Male	2 313 456	(49.2)	151 146	(6.5)	84.0	14.0	1.8	0.2	82.5	15.9	1.4	0.2
Female	2 372 196	(50.4)	141 495	(6.0)	87.6	11.0	1.3	0.1	79.1	18.9	1.8	0.2
Another	19 878	(0.4)	1917	(9.6)	80.8	16.5	2.3	0.3	71.3	24.8	3.5	0.3
Ethnicity	No.	%	n	%	%	%	%	%	No.	%	n	%
Māori	802 266	(15.0)	53 922	(6.7)	84.1	13.6	2.2	0.1	75.5	21.6	2.6	0.2
Pacific	397 920	(7.5)	19 992	(5.0)	88.7	9.4	1.4	0.5	78.9	18.1	2.5	0.6
Asian	802 434	(15.0)	18 999	(2.4)	95.5	3.8	0.5	0.2	88.6	10.3	0.9	0.2
MELAA	85 200	(1.6)	1914	(2.2)	95.0	4.3	0.5	0.1	86.7	12.0	1.1	0.2
Other	53 454	(1.0)	4812	(9.0)	81.2	16.4	2.3	0.1	76.5	21.3	2.0	0.2
European	3 199 491	(59.9)	225 594	(7.1)	83.8	14.4	1.7	0.1	79.8	18.5	1.6	0.1

*Note.* NEI (Not Elsewhere Included) respondents were excluded from this table. They comprised 16.6% of the total Census sample: Māori (29.3% hearing; 29.1% vision) and Pacific peoples (28.3% and 28.1%, respectively), which likely contributed to the underestimation of deafblindness in these groups.

### Deafblindness by Gender Identity

Gender-diverse people have the highest rates of seeing and hearing difficulties, with 9.6% affected by deafblindness, compared with 6.5% of men and 6.0% of women. They also report more hearing (16.5%) and vision (24.8%) issues than cisgender participants (Table 2).

### Ethnic Variations

Deafblindness rates differ by ethnicity: ‘Other ethnicity’ has the highest at 9.0%, followed by European (7.1%), Māori (6.7%), Pacific (5.0%), Asian (2.4%), and Middle Eastern/Latin American/African (MELAA) (2.2%). Although deafblindness is less common, Māori and Pacific peoples report higher rates of hearing and vision difficulties. For example, 13.6% of Māori and 9.4% of Pacific respondents have trouble hearing, while 21.6% of Māori and 18.1% of Pacific respondents have difficulty seeing. Pacific peoples also have the highest rate of complete blindness at 0.6%, indicating individual sensory impairments are more prevalent in these communities (Table 2).

### Geographic Distribution

Deafblindness rates are higher in rural DHB regions like West Coast (9.5%), Whanganui (8.9%), and Tairāwhiti (8.4%), while urban areas such as Auckland (4.3%) report the lowest rates (Table 3).

**Table 3.** Geographic Distribution of Deafblindness.

			Difficulty hearing						Difficulty seeing			
	Total		Deafblind		None	Some	A lot	Cannot do	None	Some	Alot	Cannot do
DHB	No.	%	n	%	%	%	%	%	%	%	%	%
Northland	182 418	(3.9)	15 195	(8.3)	80.1	17.2	2.6	0.2	75.8	21.6	2.3	0.3
Waitematā	594 135	(12.6)	28 809	(4.8)	89.1	9.7	1.1	0.1	83.7	14.8	1.3	0.1
Auckland	442 695	(9.4)	19 149	(4.3)	90.4	8.4	1.0	0.2	83.9	14.5	1.4	0.2
Counties Manukau	544 359	(11.6)	27 747	(5.1)	88.9	9.5	1.4	0.3	81.8	16.1	1.8	0.3
Waikato	410 148	(8.7)	28 593	(7.0)	84.2	13.8	1.8	0.1	79.4	18.6	1.8	0.2
Lakes	106 968	(2.3)	7698	(7.2)	82.9	14.6	2.3	0.3	77.5	19.9	2.2	0.4
Bay of Plenty	247 860	(5.3)	17 166	(6.9)	83.5	14.4	1.9	0.2	79.6	18.4	1.8	0.2
Taranaki	118 374	(2.5)	8799	(7.4)	83.0	14.9	2.0	0.1	78.6	19.4	1.8	0.1
Tairāwhiti	47 514	(1.0)	4002	(8.4)	81.2	16.1	2.6	0.1	75.6	21.6	2.6	0.2
Whanganui	63 852	(1.4)	5679	(8.9)	80.3	17.3	2.3	0.1	74.9	22.6	2.3	0.2
Hawke’s Bay	164 571	(3.5)	11 649	(7.1)	82.4	15.4	2.1	0.2	77.7	20.2	1.9	0.2
Midcentral	173 835	(3.7)	14 217	(8.2)	82.2	15.6	2.0	0.1	76.9	20.9	2.0	0.2
Wairarapa	46 863	(1.0)	3813	(8.1)	81.7	16.2	2.1	0.1	77.7	20.4	1.7	0.1
Capital and Coast	293 733	(6.2)	15 939	(5.4)	87.6	11.2	1.1	0.1	81.7	16.8	1.3	0.1
Hutt Valley	143 832	(3.1)	9471	(6.6)	85.6	12.7	1.5	0.1	79.1	19.0	1.7	0.2
Nelson/Marlborough	152 322	(3.2)	11 460	(7.5)	82.3	15.7	1.9	0.1	80.1	18.2	1.6	0.1
Canterbury	556 416	(11.8)	34 587	(6.2)	86.0	12.5	1.4	0.1	81.5	16.9	1.4	0.1
West Coast	31 767	(0.7)	3003	(9.5)	79.1	18.1	2.6	0.1	74.9	22.9	2.0	0.2
South Canterbury	57 759	(1.2)	4584	(7.9)	81.9	16.1	2.0	0.1	79.4	18.8	1.6	0.1
Southern	325 917	(6.9)	22 974	(7.0)	84.0	14.2	1.7	0.1	80.1	18.2	1.5	0.1

*Note.* NEI (Not Elsewhere Included) values are excluded from this table. NEI ranged from ~10% to 25% across regions and may contribute to the underestimation of sensory difficulties, particularly in smaller or rural DHBs with higher rates of missing data.

Hearing impairment is most prevalent in regions with higher deafblindness rates, particularly Northland (17.2%), Whanganui (17.3%), and West Coast (18.1%). Severe hearing loss is also more frequent in West Coast (2.7%), Tairāwhiti (2.7%), and Lakes (2.6%). Geographically, vision difficulties are most common in Whanganui (22.6%), West Coast (22.9%), and Tairāwhiti (21.6%), with severe vision impairment or blindness highest in Lakes (2.6%), Tairāwhiti (2.8%), and West Coast (2.2%).

## Discussion

Our findings reveal that deafblindness is a significant, under-recognised public health issue in Aotearoa, New Zealand. The data show disparities across age, gender, ethnicity, and region, highlighting the need for integrated, culturally responsive services. Measurement limitations have direct policy consequences: when deafblindness is undercounted, resource allocation, eligibility criteria, and service planning will not reflect the true burden or distribution of need, thereby reinforcing inequities for Māori, Pacific peoples, rural communities, and gender-diverse populations.

This study found that 7.4% of people (about 295 000) have both vision and hearing difficulties with correction (and are therefore considered deafblind). Deafblindness is a major, under-recognised health issue affected by age, gender, ethnicity, and location, underscoring the need for integrated, culturally responsive services.^5,11,12^ Deafblindness further limits function compared with difficulty seeing or hearing alone, increasing the risk of depression, cognitive decline, mobility issues, and social isolation.^3,5,13,14^ These outcomes illustrate Simcock’s theory of cumulative disadvantage, in which intersecting vulnerabilities intensify health inequities over time.^15^ Despite this, health systems usually address hearing and vision impairments separately, which can lead to fragmented care and delays. People with deafblindness often do not qualify for single-impairment services yet have greater needs, leaving many without adequate support.^4,13^

Studies in high-income countries show that separate sensory health systems worsen outcomes for older adults with deafblindness.^11,14^ Without integrated care, people with deafblindness experience delays in diagnosis and inadequate support. This underscores the need for tailored, intersectional services and policy for people with deafblindness.

### Age Disparities

This study shows that deafblindness increases with age, rising from 0.8% in children (5 - 14 years) to 21.6% in adults 75 years and older. Moderate impairments peak in midlife, with 7.7% reporting deafblindness, 13.3% hearing difficulty, and 25.0% vision difficulty, mainly due to age-related conditions and limited screening. This highlights the importance of mid-life sensory screenings and timely access to assistive devices and home adaptations to lessen severity.^16^ A preventive approach to capturing deafblindness aligns with the International Classification of Functioning, Disability and Health (ICF), which frames disability as influenced by impairment as well as functional, environmental, and social components.^17^ Environmental adaptations, such as improved lighting, amplified communication devices, and home modifications, can reduce barriers and boost participation among older adults who are deafblind. Recent work has highlighted differences in the functional experience and service needs of people with deafblindness between high- and low-resourced regions of the world.^6,17,18^ As Aotearoa’s population ages, sustainable aged care should include deafblind-responsive planning, as seen in Te Pae Tata, which promotes ‘ageing well’ strategies that acknowledge sensory loss.^19^

### Gender Disparities and Intersectionality

Gender disparities in deafblindness are clear: 6.5% of men, 6.0% of women, and 9.6% of gender-diverse people are affected, with the latter group also experiencing more sensory difficulties. This may reflect not only systemic inequities for LGBTQIA+ communities in health care^20^ but also population differences in access to gender-affirming treatment, comorbidities, and barriers to screening and reporting.^9,10^ The 2023 Census marked progress by introducing gender-inclusive questions,^8^ but broader reforms are needed to ensure more inclusive data collection and service provision nationwide. Health and disability services must provide gender-affirming training and remove systemic barriers through inclusive policies.^9,10^ To fulfil obligations under Te Tiriti o Waitangi and the United Nations Convention on the Rights of Persons with Disabilities (UNCRPD), services must adopt intersectional approaches that address the combined effects of gender identity, disability, and social exclusion.^21,22^

### Ethnic Inequities

Deafblindness rates show disparities and likely underreporting among Indigenous and minoritised groups. While other ethnicities (9.0%) and NZ European (7.1%) report the highest deafblind rates, Māori (6.7%) and Pacific peoples (5.0%) experience more sensory difficulties at younger ages, with high rates of vision and hearing difficulty. These trends suggest that although overall deafblind prevalence may appear lower in some ethnic groups, underlying sensory challenges are more widespread. Age-specific data show that Māori experience higher rates of hearing and vision difficulties across most age groups, indicating an earlier onset of deafblindness. This pattern aligns with evidence that Indigenous and minoritised populations are more likely to experience earlier onset of chronic conditions due to systemic factors such as poverty, racism, environmental stress, and limited access to preventive care.^11,23-25^ In Aotearoa, colonisation and systemic racism have contributed to elevated rates of conditions linked to sensory loss, such as diabetes, cardiovascular disease, otitis media, and stroke, among Māori and Pacific peoples.^26^ However, the lower reported prevalence of deafblindness in these groups is likely a methodological artefact, influenced by younger age structures and cultural underreporting. Without addressing these biases, national data risk underestimating the actual burden of early-onset sensory impairment in Indigenous communities. Standard disability measures like the WGSS may not fully capture deafblind in Indigenous and collectivist communities. In te ao Māori, disability is understood relationally, with concepts such as tāngata whaikaha emphasising collective strength and whānau support. This framing can lead to under-identification when familial care offsets functional limitations.^24,25^ Similarly, Pacific models like Fonofale prioritise spiritual, familial, and communal well-being, which may obscure impairments in individual-focused assessments.^27^

### Geographic Variations and Access Inequalities

Geographic disparities in deafblindness reflect broader structural inequities. Urban areas such as Auckland (4.3%) and Waitematā (4.8%) report the lowest prevalence, while rural and socioeconomically disadvantaged regions, including the West Coast (9.5%), Whanganui (8.9%), and Tairāwhiti (8.4%), show the highest rates. Older population profiles, limited access to health care, and poorer living conditions in rural areas contribute to these patterns. Rurality compounds inequities through barriers such as long travel distances, limited access to specialists, and delayed care,^28^ reinforcing geography as a key social determinant of deafblind risk and service access. Recognition of these disparities is growing. Te Pae Tata identifies rural and disabled populations as priority groups for improved access to services.^19^ Proposed health reforms aim to reduce geographic health disparities, aligning with Article 25(e) of the UNCRPD, which guarantees equitable health care regardless of location.^22^ Addressing these gaps requires systemic changes to health infrastructure and service delivery. Mobile and community-based services, such as sensory screening clinics, telehealth audiology, and travel support for rural residents, are essential for improving access. Training local health workers, including nurses, to conduct basic sensory assessments can support early identification and intervention. Services must also be culturally responsive, incorporating New Zealand Sign Language, tactile signing, and accessible formats such as audio and braille.

### Measurement Limitations

In public health planning, measurement determines visibility, eligibility, and resourcing. If deafblindness is undercounted or misclassified, service need will be systematically underestimated, especially for groups already underserved.

Using a broader threshold, some difficulty or more, enhances sensitivity and captures underserved populations, but reduces specificity and complicates service planning. Moreover, the WGSS’s Western framing does not align with Māori and Pacific worldviews, which emphasise relational well-being and collective care. This cultural mismatch may lead to underreporting and misclassification, particularly among communities where disability is not viewed through an individualistic lens.

To improve the accuracy and equity of disability data in Aotearoa, New Zealand data collection tools must be co-designed with disabled, Māori and Pacific communities. While a stratified classification system, such as the WGSS, offers international comparability, it does not fully capture the relational and collective understandings of disability embedded in Indigenous worldviews. A more inclusive approach would integrate functional and clinical measures with cultural models of health that reflect whānau roles, spiritual well-being, and community support. Combining these perspectives with clinical and functional assessment could enable a more nuanced analysis while maintaining alignment with global standards. Te Pae Tata endorses this approach and aligns with Article 31 of the UNCRPD, which mandates the collection of disaggregated data to uphold the rights of disabled people.^19,22^

### Policy Implications and Future Directions

These measurement limitations have direct policy consequences: when deafblindness is undercounted, resource allocation, eligibility criteria, and service planning will not reflect the true burden or distribution of need, reinforcing inequities for Māori, Pacific peoples, rural communities, and gender-diverse populations.^9^

This study highlights the need for equity-focused policy responses to address the high prevalence of deafblindness in Aotearoa, New Zealand, particularly among older adults and across ethnic and gender-diverse populations. Recent developments in health governance, such as Te Pae Tata: The Interim New Zealand Health Plan 2022,^19^ prioritise disabled people in planning and offer a strong foundation for action. To ensure meaningful inclusion, people with deafblindness must be recognised as a distinct disability group with tailored services that meet their specific communication, mobility, health, and social needs.

Integrating deafblindness into national disability strategies could be achieved through the New Zealand Disability Strategy^29^ or the development of a dedicated Deafblindness Action Plan. Internationally, organisations such as the World Federation of the Deafblind warn that failing to recognise Deafblindness as a distinct disability leads to invisibility in data, policy, and services.^30^ Establishing a national deafblindness working group under the could support culturally relevant definitions, improve cross-sector coordination, and strengthen data collection. This aligns with Article 4(3) of the UNCRPD,^22^ which mandates the active involvement of disabled people in policy design and implementation, while co-designing systems with deafblind people and their whānau ensures cultural relevance, responsiveness, and legitimacy.

Addressing systemic inequities in deafblindness services requires alignment with Te Tiriti o Waitangi and the UNCRPD. The principle of ōritetanga (equity) affirms that Māori must receive services proportionate to their higher needs. This includes greater investment in preventive interventions for sensory impairment, such as early diabetes management and accessible audiology, particularly within Māori and Pacific communities. The WAI 2575 Health Services and Outcomes Inquiry identified ongoing breaches of Te Tiriti o Waitangi due to inequitable primary care provision. Deafblindness services must be designed to avoid repeating these patterns and instead work to actively redress them.^21,22^

Articles 9, 21, and 25 of the UNCRPD affirm the rights of disabled people to accessible environments, communication support, and community-based health care. In practice, this means expanding services, for example, NZ Relay, increasing access to dual-mode communication (eg, tactile signing and captioning), and implementing auditory and visual alert systems in health care settings to ensure accessibility for people with deafblindness.

Culturally responsive, holistic services should be standard. Indigenous models like Te Whare Tapa Whā and Fonofale highlight the interconnectedness of well-being. Applying these to Deafblindness services could include whānau-based care plans, cultural reconnection for deafblind individuals, and community delivery through marae or churches. These approaches honour the collective, relational nature of disability in Māori and Pacific communities, ensuring accessibility and cultural safety.

Frameworks such as the Atoatoali’o National Pacific Disability Approach alongside Māori health strategies highlight the need for culturally competent care. Building a disability workforce aligned with these frameworks involves training in culturally grounded communication, recognising relational views of disability, and embedding Indigenous values into service design.

## Conclusions

This study highlights disparities in deafblindness across Aotearoa, emphasising the need for an equity-focused response. Current tools often overlook the complex, culturally nuanced nature of deafblindness, especially among Māori, Pacific peoples, and rural communities. Recognising deafblindness as a distinct disability and co-designing services with affected communities is vital. Aligning with Te Tiriti o Waitangi and the UNCRPD, and integrating Indigenous well-being models, will help create more inclusive and responsive support systems for deafblind people.
